# Annual Meeting of Governors and General Council

**Published:** 1915-04-24

**Authors:** 


### KING EDWARD'S HOSPITAL FUND FOR LONDON.

### Annual Meeting of Governors and General Council.

The annual meeting of the Governors and General Council of King Edward's Hospital Fund for London, to receive the accounts and the report of the General Council for the year 1914, was held at St. James's Palace on April 19, the Speaker of the House of Commons being in the chair.

Those present were: The Viscount Iveagh, the Viscount Knutsford, Lord Sandhurst, Lord Revelstoke, Lord Farquhar, Lord Stamfordham, the Hon. Arthur Stanley, M.P., the Hon. H. L. W. Lawson, M.P., the Chairman of the County Council (Mr. Cyril Jackson), the President of the Royal College of Physicians (Dr. Frederick Taylor), the Right Hon. C. Stuart-Wortley, K.C., M.P., the Right Hon. Sir Savile Crossley, Bart. (Hon. Secretary), the Right Hon. Sir Vezey Strong, Sir William S. Church, Bart., Sir Henry Burdett, Sir Owen Philipps, Sir William J. Collins, Sir Horace Marshall, Sir John Craggs, Sir John Tweedy, Dr. Edwin Freshfield, Mr. John G. Griffiths (Hon. Secretary), and Mr. Walter Morrison.

### The King's Tribute to the late Lord Rothschild.

Sir Savile Crossley read the following letter from his Majesty the King through Lord Stamfordham:—

"Windsor Castle, April 12, 1915.

"Dear Sirs,—I am commanded by the King to convey to the Governors and General Council an expression of his Majesty's deep sense of the great loss which the Fund has sustained in the death of Lord Rothschild, its Honorary Treasurer and Chairman of the Finance Committee.

"It was at the request of his late Majesty King Edward VII., then Prince of Wales and President of the Fund, at the inaugural meeting of the first General Council, that Lord Rothschild undertook the duties of Treasurer. His wise counsel and judgment have been of inestimable value to the Fund, and thereby have exercised a powerful and beneficial influence upon the hospitals of London.

"His Majesty, as patron of the Fund, cordially approves the action of the Governors in appointing as Lord Rothschild's successor Lord Revelstoke, who has for many years been an active member of the Finance Committee.

"The King gratefully appreciates the services given by the Governors of the Fund, his Highness the Duke of Teck, Viscount Iveagh, and the Speaker of the House of Commons, in carrying out the duties of President during their five years' term of office, which expires in June next. Having ascertained that the Lord Chancellor, the Prime Minister, and the Governor of the Bank of England, pursuant to the Act of Incorporation of the Fund, are prepared to recommend their re-appointment, his Majesty has commanded me to inform you of his intention, until the appointment of a President, to reappoint them to the office, the duties of which they have so efficiently discharged. Yours very truly,

"(Signed) Stamfordham.

"The Honorary Secretaries,

"King Edward's Hospital Fund for London."

#### The Late Treasurer's Services.

The Speaker of the House of Commons then said: My Lords and Gentlemen,—I now have to move the following resolution:—

"That the Governors and General Council desire to place on record their profound regret at the death of Lord Rothschild, Honorary Treasurer of the Fund since its foundation. They recall with gratitude the great services rendered by him as treasurer and as an active member of the Council during the whole period of its existence, as trustee and member of the Executive Committee down to the incorporation of the Fund by Act of Parliament in 1907, and subsequently as chairman of the Finance Committee. They are deeply indebted to Lord Rothschild for the skill and care which he devoted to the ever-growing responsibilities of his office, and they deplore the loss of a colleague whose name, whose ability, and whose interest in the work of charity were a constant source of strength to the Council and to the Fund."

I think I need hardly add anything to the terms of the resolution itself, except to say, on my own behalf and on that of my fellow Governors, and I am sure I can also say it on behalf of all the members of the several Committees of the Fund, how much we shall miss the assistance and support of Lord Rothschild, and how conscious we are of the great advantage the Fund derived from having him for its Treasurer during all the years in which its present financial position was being gradually built up. The Council will doubtless desire that in forwarding this resolution I should add an expression of condolence with the family of Lord Rothschild in their bereavement.

The resolution, having been seconded by Lord Iveagh, was carried unanimously.

### The New Treasurer and Committeemen.

The Speaker of the House of Commons then said: My Lords and Gentlemen,—On the death of Lord Rothschild it became the duty of the Governors, under the terms of the Fund's Act of Incorporation, to appoint a successor in the offices of Honorary Treasurer and Chairman of the Finance Committee. Lord Revelstoke was one of the original members of the Finance Committee, and has for several years devoted the closest and most watchful attention to the care of the Fund's investments, which is the particular duty of that Committee. We are glad to say that he has consented to undertake, in addition to these responsibilities, the further duties of Honorary Treasurer and Chairman of the Committee. They could not be in better hands, and, as you have heard, his Majesty, the patron of the Fund, has been graciously pleased to express approval of the appointment.

We have also, with his Majesty's approval, made the following appointments: The Hon. Algernon H. Mills, who is already on the Finance Committee, to be a member of the Council, and the Hon. Nathaniel Charles Rothschild to be a member of the Finance Committee.

#### Satisfactory Income from Investments.

Lord Revelstoke (the Honorary Treasurer) in presenting the account of receipts and expenditure and the balance-sheet for the year ending December 31, 1914, said: It will be noticed that the additions to the capital account are largely in excess of those shown in normal years. This increase is principally accounted for by the receipt for capital account of £313,000, being the amount received on account of the one-twelfth share of residue in connection with the legacy of the late Sir Julius Wernher. The balance-sheet itself is clear, and would not seem to call for any particular comment; but it is satisfactory to be able to report that, in spite of the financial disturbance occasioned by the war, the receipt of income derived from investments continues to be quite satisfactory.

### THE COUNCIL'S REPORT.

Sir Savile Crossley (Hon. Secretary) presented the draft report of the Council for the year 1914 as follows :—

The total receipts for the year 1914 were £529,885 14s. 3d., of which £315,093 10s. 11d. were contributions to capital, and £214,792 3s. 4d. receipts on general account. The general fund receipts were made up as follows: Annual subscriptions, £25,876 2s. 5d.; donations, £5,371 19s. 1d.; contribution from the League of Mercy, £14,000; from the Lewis Estate, £30,863; Harman Bequest, ground rents valued at, £25,000; other legacies, £28,496 9s. 3d.; interest from investments, £84,984 12s. 7d.; from the Trustees of the Bawden Fund, £200; making a total of £214,792 3s. 4d.

A Conservative Policy.

In this year of war the amount distributed was £140,000, as against £157,500 in the previous year. Although the total distributed was less than in 1913, there was an increase in grants in aid of the ordinary maintenance of hospitals, the reduction being wholly accounted for by a diminution in the claims on the Fund for building expenditure. This diminution was due to the postponement by the hospitals at the outbreak of the war of all schemes not actually in hand or particularly urgent. Of the sum of £140,000 distributed the hospitals received £133,500, the remaining £6,500 being distributed amongst consumption sanatoria and convalescent homes taking London patients.

The £133,500 allotted to the London hospitals was directed to be applied as follows: £108,550 to general purposes and maintenance; £12,600 towards the liquidation of debts on maintenance account; and £12,350 for additions and improvements to hospitals and their equipment. Of the £6,500 given to consumption sanatoria and convalescent homes, £3,800 was allotted in consideration of the allocation of beds for patients to be sent from certain London hospitals.

### Decrease in Administration Expenses.

During the year the amount spent on administration was 12s. per cent. of the total amount received, as compared with £1 12s. 5d. last year. The corresponding percentage for the whole period since the inauguration of the Fund has been £1 4s. 5d.

His Majesty the King, patron of the Fund, has been graciously pleased to continue his annual subscription of £1,000. Her Majesty Queen Mary, Her Majesty Queen Alexandra, His Royal Highness the Prince of Wales, and many other members of the Royal Family have also again honoured the Fund with their support.

The list of contributions includes annual subscriptions of £5,000 from Mr. Walter Morrison, £4,000 from the Drapers' Company, £1,000 from Mr. William Waldorf Astor, and £1,000 from the Merchant Taylors' Company; besides two anonymous donations of £1,000. The Fund has also received annual subscriptions of £500 from the City Corporation, from Lord Iveagh, from Mr. Robert Fleming, from Messrs. Morgan, Grenfell and Co., and from Messrs. N. M. Rothschild and Sons; and donations of the same amount from the Clothworkers' Company, from "G. C.," and from "S.D.R.S.D."

Substantial Benefit from the League of Mercy.

The contribution of the League of Mercy this year was again £14,000, a result which reflects great credit on the efforts of the members of the League to secure recognition of the claims of the hospitals at the present time. The total amount received from the League of Mercy since its foundation in 1899 now amounts to £216,000, apart from the sums distributed by the League to institutions outside the area of the Fund's distribution. The Council desire to express their appreciation of this very substantial benefit derived from the efforts of a numerous band of voluntary workers.

Legacies received during the year include (in addition to the Lewis and Harman bequests) £9,027 12s. 3d., residue of the estate of the late Mr. John Harold Goodwin; £8,070 further on account of the residue of the estate of the late Mr. George Hunt Heigham; £2,323 14s. 6d., balance of residue of the estate of the late Mr. Edward Matyear; and £2,232 1s. 2d., a share in the residue of the estate of the late Mr. Peter Meech.

The amount announced in the last annual report as having been received in February 1914 from the estate of the late Sir Julius Wernher was increased by further receipts during the year to £313,715 13s. 2d. This sum represents the first instalments of the very generous legacy of one-twelfth of the residue of his estate, left in augmentation of the capital account of the Fund. This bequest from a member of the Council of the Fund is one of the most important of the munificent gifts to capital which have done so much to enable the annual distribution to be increased to the level attained in recent years.

### Requirements for 1915.

The sum required to be raised during the year, after taking into account income from investments, in order to maintain the distribution of £140,000, is nearly £60,000; while to reach again the distribution of the three previous years more than £75,000 must be obtained in the year by means of annual subscriptions, donations, and legacies.

The total amount received from annual subscriptions and donations during 1914 showed, as compared with 1913, a decrease of £750, while the receipts from legacies (other than the Lewis and Harman bequests) were £19,525 less than the previous year, or £6,223 less than the average of the five years 1909 to 1913.

The nation will surely realise that the work done by the hospitals, both in the treatment of the wounded and in the providing and training of doctors, nurses, and administrators in the Medical Service of the Army and Navy, gives them a special claim on the generosity of the public. The Council trust, therefore, that notwithstanding the increased demands made by the war and by special funds for purposes arising out of the war, the needs of the hospitals, and the importance of maintaining the annual distribution of the Fund, will continue to be recognised.

The annual statistical report prepared by the Fund on the expenditure of 106 London hospitals again shows a general increase in cost of working. Though this is attributable partly at least to the rise in prices and the development of new and more expensive treatments, the Council have in the remarks accompanying their grants continued to draw attention to any instances of high or increasing cost which appear to require further explanation. The Council believe that by preparing comparative figures, and by thus encouraging economy of management, the Fund is assisting the hospitals not only in their ordinary work, but in reducing as far as possible the losses unavoidably caused by high prices and other difficulties during the war.

### The Revised Uniform System.

The revised Uniform System of Accounts has during the year been reissued in a new edition. Since its original preparation in 1906 by this Fund, in co-operation with

### THE COUNCIL'S REPORT (*continued*).

the Hospital Sunday and Hospital Saturday Funds and in consultation with a Committee of Hospital Secretaries, the rules of the system have been supplemented by several memoranda embodying new regulations arising out of the application of the system to the circumstances of different institutions. In the new edition all the various detailed regulations are collected together in a codified form, with a short summary of the general principles underlying them. It is believed that this arrangement will greatly facilitate the use of the system which has become the recognised standard of hospital account-keeping.

The Council view with great satisfaction the successful amalgamation of the Hospital for Diseases of the Throat, Golden Square, and the London Throat Hospital, Great Portland Street. The Fund has allocated to this scheme out of the amounts promised or set aside in aid of a general amalgamation of the throat hospitals of London a capital grant of £10,000 and a maintenance grant of £900 a year for five years, subject to the approval of the proposed building scheme and to the usual annual inspection by the Fund's visitors.

### Pensions for Hospital Officers.

During the year the Executive Committee, as the result of representations made to the Fund some time ago by the Hospital Officers' Association, undertook an inquiry into the question of pensions for hospital officers. The following were appointed members of a sub-committee for the purpose: Mr. W. J. H. Whittall (chairman), Sir William Collins, and Mr. Hopkinson. This inquiry was not completed when the Council for 1914 laid down its duties.

Each eligible hospital and consumption sanatorium which applied for a grant was inspected and reported upon by two of the Fund's visitors, one medical man and one layman in each case.

The Convalescent Homes Committee have again secured by means of grants from the Fund the reservation of forty-four beds at five consumption sanatoria, and have placed them at the disposal of London hospitals for the accommodation of patients urgently needing country treatment.

#### Hospital Extension and Reconstruction.

The Distribution Committee have carefully considered all schemes for the extension or reconstruction of hospitals which have been submitted in accordance with the request of the Council, though the number thus submitted has been less than usual owing to the war. The Fund has thus used its best endeavours to assist the hospital authorities in ensuring that when capital expenditure is undertaken adequate consideration is given to economy, efficiency, and prospect of funds for future maintenance; and has particularly considered the relation of each scheme to the general question of hospital accommodation in London.

#### Deaths and Resignations.

The Council regret to record the deaths of Sir R. Lucas-Tooth, a member of the Council and a generous donor to the fund; of Mr. S. H. Benson, who had been a member of the Organising Committee of the Coronation Gift to King Edward VII., and successively of the Visiting, Executive, and Convalescent Homes Committees; and also of Mr. Reginald Lucas, who had been a visitor for many years. To the lamented death in 1914 of Lord Strathcona, one of the greatest benefactors of the Fund and a member of the Council, full reference was made in the last annual report.

The Council much regret that Mr. William Latham, K.C., has been compelled through ill-health to resign the chairmanship of the Convalescent Homes Committee, an office which he had held since 1898, and in which he is succeeded by Dr. Edwin Freshfield. They also regret the retirement of Mr. A. G. Sandeman, who had been a member of the Council since the foundation of the Fund.

The President of the Local Government Board, in pursuance of his powers under the Act, has appointed Messrs. Deloitte, Plender, Griffiths and Co. to audit the accounts of the Fund for the year.

The Council are under great obligations to the Press for the valuable services they have rendered in the past, and continue to rely upon their kindly co-operation in obtaining for the Fund wider publicity and support.

### The Speaker's Address.

The Speaker of the House of Commons, in moving the adoption of the report, said: My Lords and gentlemen, it is not the practice of the presiding Governor when moving the adoption of the annual report at this meeting to make a long speech, but only to refer to any special point that arises out of the accounts or the report.

With regard to the accounts, a few minor alterations in the form of the statement of receipts and expenditure and of the balance-sheet have been introduced for the purpose of making the transactions of the twelve months rather more clear to the general reader. I should like to take this opportunity of thanking our auditors, Messrs. Deloitte, Plender, Griffiths and Co., for the very valuable services they have rendered to the Fund in connection with the accounts since its foundation.

The Council will also agree with me that we are very fortunate, in these times especially, in having our invested funds in the safe keeping of so competent a Finance Committee as that over which Lord Rothschild presided for so many years, and over which Lord Revelstoke will now preside.

#### Throat Hospitals Amalgamation.

One of the most gratifying incidents of the past year was the completion of the amalgamation of two of the throat hospitals, the Hospital for Diseases of the Throat, Golden Square, and the London Throat Hospital, in Great Portland Street. The amalgamation of small hospitals, where there seems to be no special reason for continuing their separate existence, involving duplicate establishments, has long been favoured by this Fund; and this is particularly the case where new building schemes are contemplated.

In the present instance the expiration of the lease of the London Throat Hospital coincided with the acquisition of space for the extension of the Golden Square Hospital. This amalgamation has, therefore, every form of advantage. The patients and the medical staff will benefit by the improvements which are more easily introduced in a larger hospital; economy of management will be facilitated by the concentration of the work in a single establishment; and there will be only one building scheme instead of two. The chairmen, committees of management, and medical staffs of the hospitals concerned are to be congratulated on their action; and a share in the credit may, I think, fairly be claimed by this Fund for the assistance and encouragement it has given both in the consideration of the amalgamation proposals by the Distribution Committee and in the substantial grants made in aid of the scheme. In so doing the Fund has only been continuing the policy which had proved successful in the past, in the case of the Orthopædic Hospitals and of the Hampstead and North-West London Hospitals; a policy which has, since the end of the year, again been justified in the amalgamation, under the auspices of the Charity Commissioners, and with the support of the Fund, of the British Lying-in Hospital with the Home for Mothers and Babies, in Woolwich. I now have the honour to move the adoption of the report.

The President of the Royal College of Physicians (Dr. Frederick Taylor) seconded the motion, which was carried unanimously.

The Rt. Hon. C. Stuart-Wortley, K.C., M.P., proposed a vote of thanks to the Speaker of the House of Commons for presiding. The resolution was seconded by the Hon. Arthur Stanley, M.P., and carried unanimously.

The Speaker having briefly acknowledged the vote of thanks, the proceedings terminated.

